# Left hepatic abscess with diaphragm involvement in the setting of biliary ostomy

**DOI:** 10.1093/jscr/rjaf224

**Published:** 2025-04-14

**Authors:** Grant H Gershner, Catherine J Hunter, Morgan Bonds

**Affiliations:** Department of Surgery, The University of Oklahoma Health Sciences Center, 800 Research Parkway, Suite 449, Oklahoma City, OK 73104, United States; Division of Pediatric Surgery, Oklahoma Children’s Hospital, 1200 Everett Drive, ET NP 2320, Oklahoma City, OK 73104, United States; Department of Surgery, The University of Oklahoma Health Sciences Center, 800 Research Parkway, Suite 449, Oklahoma City, OK 73104, United States; Division of Pediatric Surgery, Oklahoma Children’s Hospital, 1200 Everett Drive, ET NP 2320, Oklahoma City, OK 73104, United States; Department of Surgery, The University of Oklahoma Health Sciences Center, 800 Research Parkway, Suite 449, Oklahoma City, OK 73104, United States

**Keywords:** partial biliary diversion, biliary ostomy, hepatic abscess

## Abstract

This case report discusses the indications, surgical technique, and outcomes associated with a biliary ostomy in a patient with complex biliary tract disease, providing insights into its role in modern surgical practice.

## Introduction

External biliary drainage is a crucial therapeutic approach in the management of biliary diseases. Common methods are done via endoscopy or interventional radiology [1–3], which are preferred due to their lower morbidity and quicker recovery times. In certain complex cases, definitive surgical approaches may be required. External biliary diversion can effectively relieve biliary pressure and prevent complications like cholangitis or biliary cirrhosis. Although it is a well-established procedure, its use has fallen out of common practice due to minimally invasive techniques. This manuscript highlights the persistence of traditional biliary drainage methods in select complex cases.

## Case report

**Figure 1:**
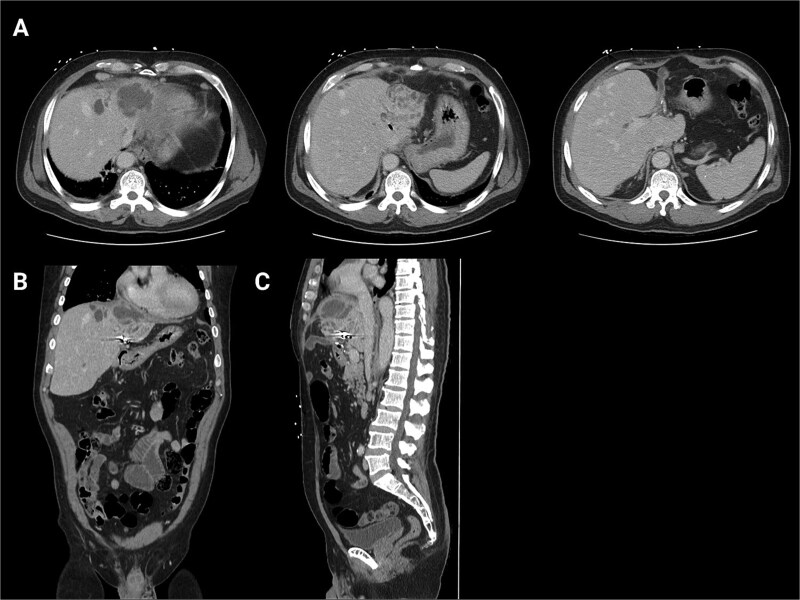
Initial CT scans. (A) Axial images progressing from superior to inferior. These show the hepatic abscess as well as the small bowel limb anastomosed to the biliary tree. (B) The same scan, but in a coronal view. (C) The same scan, but from a sagittal view. This view again illustrates the limb of a small bowel anastomosed to the biliary tree. Surgical clips are seen in the gall bladder bed.

A 62-year-old male presented with the chief complaints of fevers, fatigue, and body aches for one week. He reports an ostomy placed fifteen years prior to, “drain the rocks from my liver.” He reports that stones drained from the ostomy for two years after it was placed. Physical exam was notable for abdominal incisions and left upper quadrant ostomy. Initial workup found a WBC count of 14 000×10^3^/μl, alkaline phosphatase of 571 U/L, and normal bilirubin. Given his vague abdominal pain and fevers, a contrasted computed tomography (CT) scan of the abdomen was performed. This confirmed that the ostomy was a conduit from the biliary tree to the skin. It additionally found a 6.6 × 6.4 cm intrahepatic abscess (Fig. 1). He was admitted and was started on IV antibiotics. Gastroenterology (GI) and Interventional Radiology (IR) were consulted for endoscopic or image-guided drainage, but these were deferred given the tortuosity of his conduit and lack of a safe window, respectively. Given he had improved on antibiotics, it was deemed that operative intervention was not indicated at that time. He was transitioned to oral antibiotics, scheduled for outpatient endoscopic retrograde cholangiopancreatography (ERCP) to better delineate the biliary anatomy, and discharged. Of note, this institution’s policy is that Advanced Endoscopists perform ERCPs. At this time, there are no surgeons who performed ERCPs.

**Figure 2:**
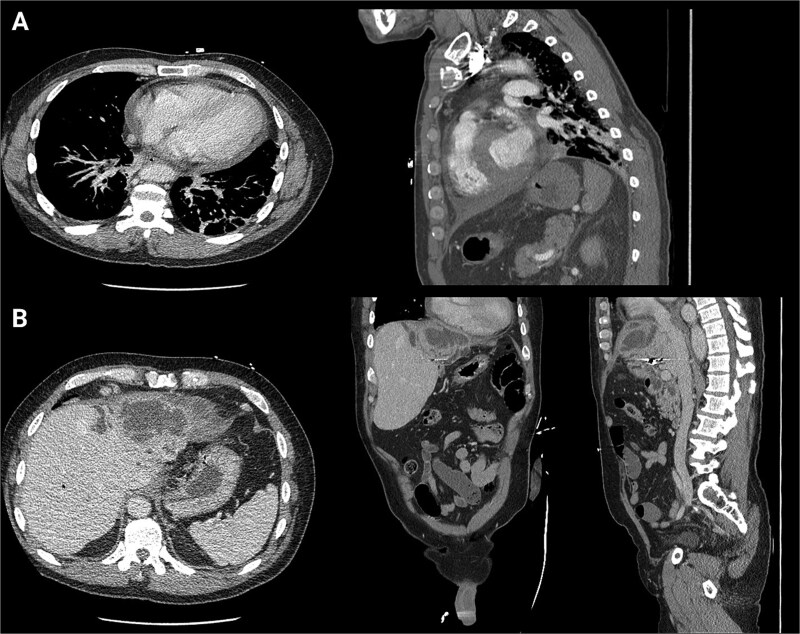
First readmission CT scans. (A) CTA chest images in the axial and sagittal planes illustrating the newly developed pericardial effusion. (B) CTA abdomen/pelvis images in the axial, coronal, and sagittal planes. These illustrate the abscess increasing in size.

**Figure 3:**
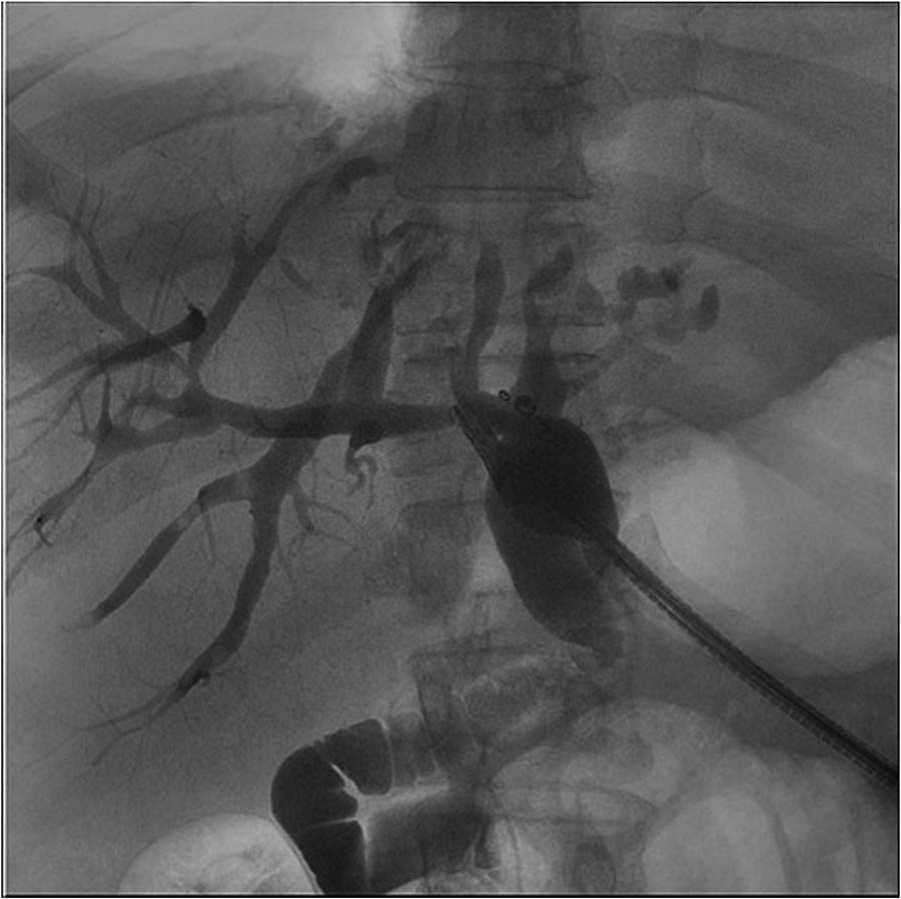
Fluoroscopic image from ERCP—a representative image from the patient’s ERC. This demonstrates biliary ductal dilation but no signs of obstructing stones or stricture. Additionally, this image illustrates the cystic cavity does not communicate with the biliary system.

The patient presented the following day with fevers and new chest pain. He underwent a computed tomography angiography (CTA) chest and abdomen/pelvis, which revealed a left lower lobe consolidation and a moderate pericardial effusion. His hepatic abscess had increased in size to 7.2 × 5.5 cm (Fig. 2). Cardiology was consulted, and a bedside echo was performed, which found acute pericarditis. He was admitted and started on IV antibiotics and colchicine for his pericarditis. GI subsequently performed an ERCP, which showed no biliary or anastomotic stricture (Fig. 3). It was thought that it was likely a choledochal cyst, and the plan was for elective left hepatectomy. He was transitioned to oral antibiotics and discharged with colchicine for 3 months.

**Figure 4:**
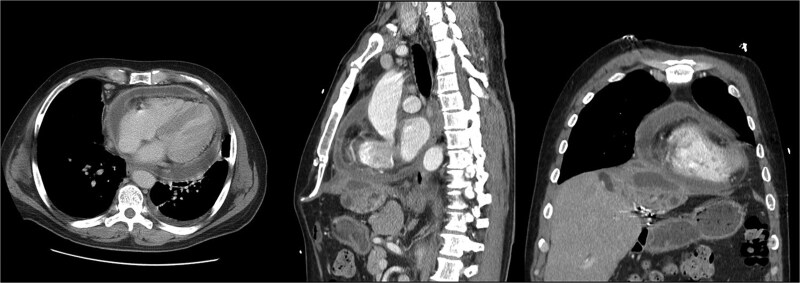
Second readmission imaging—CT of the chest in the axial, sagittal, and coronal views. These demonstrate that the abscess has eroded through the diaphragm and now abuts the pericardium. Additionally, it shows a worsening of the pericardial effusion, concerning for abscess communication.

He was seen in the clinic 6 days later. At that time, he reported worsening abdominal pain and orthopnea. Repeat imaging showed that the hepatic abscess was stable in size, but the pericardial effusion had worsened and that the hepatic abscess had broken through the right hemidiaphragm (Fig. 4). Given his multiple instances of failed management, HPB Surgery elected to perform diagnostic laparoscopy. A TTE was performed, which showed a loculated pericardial effusion. Cardiothoracic Surgery (CTS) was consulted to see if drainage was indicated. They deemed that a pericardial window was needed, but it would depend on the outcome of the hepatic abscess drainage.

### Surgical intervention

A Hassan technique was used to enter the abdomen, but dense adhesions were encountered, which precluded safe entry. A sub-xiphoid mini-laparotomy was then performed. Near the falciform, an area of dense adhesions and inflammation was noted between the liver, diaphragm, and pericardium. This was dissected until purulent material was expressed. This was suctioned and sent for cultures. Since the patient had no stones draining from his ostomy, it was decided to take down his ostomy as well.

### Postoperative course

He recovered well postoperatively. A repeat echocardiogram showed that his effusion had decreased in size, so CTS deferred the window. His hepatic cultures were positive for *Cutibacterium acnes*, and Karius testing was positive for *Mycobacterium mucogenicum* and *Pseudomonas*. ID recommended an oral regimen of levofloxacin, metronidazole, and azithromycin. He was subsequently discharged. As of writing, the patient is doing well.

## Discussion

### Hepatic abscesses

In North America, hepatic abscesses are fairly uncommon, with an incidence of 2.3 cases per 100 000 admissions [4]. Hepatic abscesses are effectively managed with percutaneous 95% [5] or endoscopic drainage 100% [6]. General indications for surgical drainage of hepatic abscesses include failure of or lack of percutaneous capabilities, abscesses >5 cm, or multiloculated abscesses [7].

### External biliary diversion

**Figure 5:**
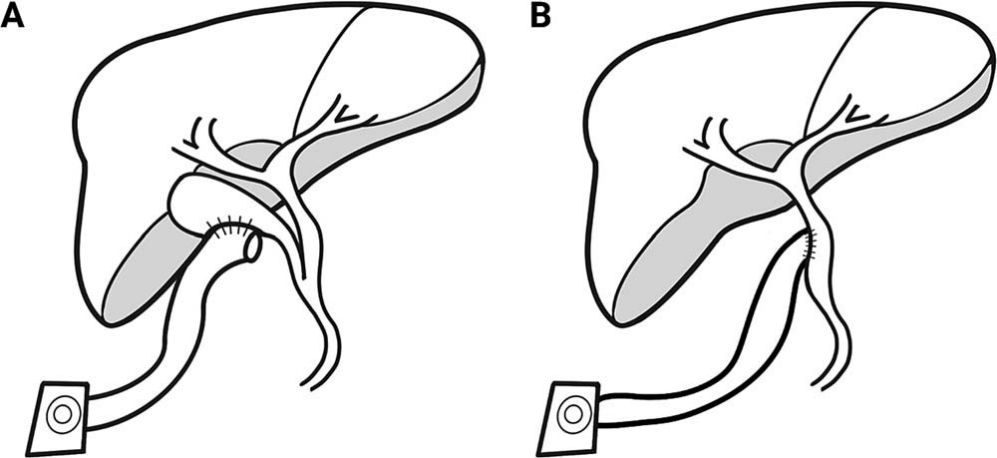
PEBD diagram. (A) A diagram representing the typical post-surgical anatomy for PEBD. This includes isolating a small bowel conduit and anastomosing this to the gallbladder. The other end is brought out through the skin to form an ostomy that will drain bile and stones. This is commonly done in children with chronic biliary stasis. (B) A diagram of the anatomy encountered in this case. The anatomy is similar, except the gallbladder is surgically absent, and the conduit is anastomosed directly to the biliary tree.

Although surgical external biliary drainage is a known operation, it is not commonly encountered. Discussing this case with multiple faculty found no usage within the past several decades. Attempts to obtain his history from the original hospital/surgeon were unsuccessful. After the literature review, the operation that resembles what the patient had is partial external biliary diversion (PEBD). This procedure is most often seen among pediatric surgeons, as it is used to treat children with chronic intrahepatic cholestasis from syndromes like progressive familial intrahepatic cholestasis. This is described as using a segment of the small bowel as an interposition between the gallbladder and the skin [8] (Fig. 5). A key difference for our patient is that our patient’s gallbladder was surgically absent, and his anastomosis was connected directly to the common bile duct. Additionally, this operation is typically done at a young age (between 1 and 13, [9]). Our patient had his surgery done at the age of 47. This patient’s procedure was also performed in 2009, by which ERCP had become widely available. Since we are unable to obtain the records for this patient, the exact indications and decision-making that led to him undergoing this modified PEBD are unknown.

## Conclusion

In conclusion, while external surgical biliary drainage techniques have fallen out of favor in modern practice due to the rise of minimally invasive alternatives, they can still be performed in select patients. This case illustrates that the role of surgery remains indispensable in the management of complex biliary disease or for complications.

## References

[1] Covey AM, Brown KT. Percutaneous transhepatic biliary drainage. Tech Vasc Interv Radiol 2008;11:14–20. 10.1053/j.tvir.2008.05.003. 18725138

[2] Dumonceau JM . Biliary ERCP. Endoscopy 2008;40:50–4. 10.1055/s-2007-967044. 18058618

[3] Salerno R, Davies SEC, Mezzina N, et al. Comprehensive review on EUS-guided biliary drainage. World J Gastrointest Endosc 2019;11:354–64. 10.4253/wjge.v11.i5.354. 31205596 PMC6556484

[4] Lin Y-T, Liu C-J, Chen T-J, et al. Pyogenic liver abscess as the initial manifestation of underlying hepatocellular carcinoma. Am J Med 2011;124:1158–64. 10.1016/j.amjmed.2011.08.012. 22114829

[5] Haider SJ, Tarulli M, McNulty NJ, et al. Liver abscesses: factors that influence outcome of percutaneous drainage. Am J Roentgenol 2017;209:205–13. 10.2214/AJR.16.17713. 28504550

[6] Singhal S, Changela K, Lane D, et al. Endoscopic ultrasound-guided hepatic and perihepatic abscess drainage: an evolving technique. Therap Adv Gastroenterol 2014;7:93–8. 10.1177/1756283X13506178. PMC3903087 24587822

[7] Lardière-Deguelte S, Ragot E, Amroun K, et al. Hepatic abscess: diagnosis and management. J Visc Surg 2015;152:231–43. 10.1016/j.jviscsurg.2015.01.013. 25770745

[8] Sahloul A, Lainka E, Kathemann S, et al. Progressive familial intrahepatic cholestasis—outcome and time to transplant after biliary diversion according to genetic subtypes. Front Surg 2023;10:10. 10.3389/fsurg.2023.1074229. PMC10287053 37361697

[9] Bjørnland K, Hukkinen M, Gatzinsky V, et al. Partial biliary diversion may promote long-term relief of pruritus and native liver survival in children with cholestatic liver diseases. Eur J Pediatr Surg 2020;31:341–6. 10.1055/s-0040-1714657. 32707578

